# Correction: Synthesis of an exfoliated kaolinite–poly(urea–formaldehyde) nanocomposite

**DOI:** 10.1039/d5ra90012c

**Published:** 2025-02-10

**Authors:** Hervé Barye Tatang, Jacques Richard Mache, Cyrill Joël Ngally Sabouang, Angelina Razafitianamaharavo, Renaud Gley, Sakeo Kong, Jean Aimé Mbey

**Affiliations:** a Laboratory of Applied Inorganic Chemistry, Department of Inorganic Chemistry, University of Yaoundé I P. O. Box 812 Yaoundé Cameroon mbey25@yahoo.fr jean-aime.mbey@facsciences-uy1.cm barye.tatang@facsciences-uy1.cm; b School of Geology and Mining Engineering, University of Ngaoundere P.O. Box 115 Meiganga Cameroon; c Departments of Chemistry, Higher Teacher Training College, University of Bamenda P. O. Box 39, Bambili Cameroon; d Université de Lorraine, CNRS, LIEC F-54000 Nancy France

## Abstract

Correction for ‘Synthesis of an exfoliated kaolinite–poly(urea–formaldehyde) nanocomposite’ by Hervé Barye Tatang *et al.*, *RSC Adv.*, 2025, **15**, 3026–3039, https://doi.org/10.1039/D4RA08707K.

The authors regret that an incorrect version of eqn (2) and (3) were included in the original article. The correct version of eqn (2) and (3) are presented here.

Eqn (2):
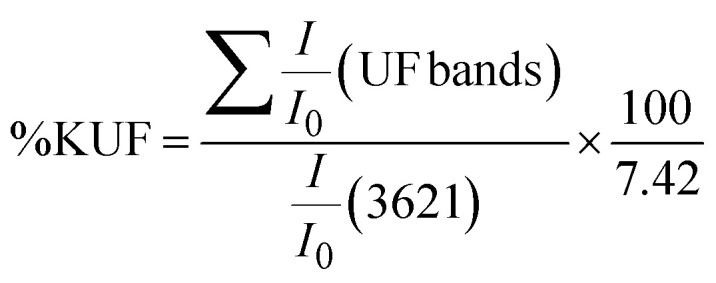


Eqn (3):
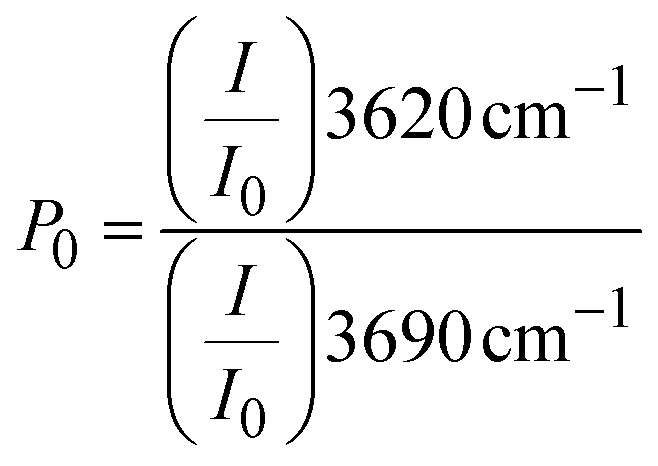


The Royal Society of Chemistry apologises for these errors and any consequent inconvenience to authors and readers.

